# Cardiovascular Disease Prevalence in Asians Versus Americans: A Review of Genetics, Diet, and the Call for Enhanced Prevention and Screening

**DOI:** 10.7759/cureus.58361

**Published:** 2024-04-16

**Authors:** Jeevan Y Nammi, Roshini Pasala, Susnigdha Kotaru, Swetha Sree Bandikolla, Nikhil Andhe, Praneeth Reddy Gouravaram

**Affiliations:** 1 Medicine, Siddhartha Medical College, Vijayawada, IND; 2 Medicine, NRI Institute of Medical Sciences, Visakhapatnam, IND

**Keywords:** apolipoprotein, genetic variation, americans, south asians, cardio vascular disease

## Abstract

Cardiovascular disease (CVD) continues to pose a global health challenge, demonstrating significant disparities in occurrence among various populations. A wide number of research studies have indicated a higher prevalence of cardiovascular disease in South Asian immigrants compared to the local American population. The demand to improve the cardiovascular benefits of immigrants is increasing, which calls for further research with larger and more diverse population samples. This study will investigate the major causes of this variation, which include genetically diverse characteristics and changes in nutritional status among the study population groups.

To assess the increase in the prevalence of cardiovascular disease among South Asian populations compared to the US population, a narrative review of accessible data is carried out. The data in support of the present document are from the Centre for Disease Prevention and Control, Statistics for Heart Diseases and Stroke 2023, a trend analysis about incidences of cardiac diseases and global burden in 2017, all dating back to the last two decades. Relevant articles from PubMed and Google Scholar have also been included, as appropriate, and their references are provided wherever necessary. Graphs for the geographical variations in disease incidence are produced using Microsoft Excel (Microsoft® Corp., Redmond, WA).

The review shows that there is a significant decline in the prevalence of CVD among American citizens when compared to the steady increase in the number of cases among South Asians, which is attributed to the unique genetic predisposition of South Asians to be more prone to CVDs. The changing dietary habits also play an important role in the fall in HDL levels in South Asians when compared to Americans. This is driven by genetic disparities, including the APOA1 and APOA2 genes, and nutritional disparities, including variance in quality and quantity of dietary consumption. Addressing the escalating cases of CVD among South Asians necessitates additional research to enhance proactive preventive measures and implement screening programs specifically tailored to address prevalent risk factors within the population.

## Introduction and background

Cardiovascular disease (CVD) is a group of conditions that affect the heart and blood vessels and pose a significant global health challenge. These conditions include coronary artery disease, heart failure, stroke, and hypertension, among others. CVD is a leading cause of mortality worldwide [1], and understanding its risk factors, prevention, and management is important.

CVD prevalence can vary significantly across different ethnicities due to a variety of factors, including genetic predisposition, lifestyle choices, socioeconomic status, access to healthcare, and cultural factors. It has been a significant health concern among Caucasian populations, particularly in Western countries. However, due to advancements in medical care and public health initiatives, the prevalence of CVD has been decreasing in many Caucasian populations. African Americans tend to have higher rates of CVD compared to Caucasians. They are more likely to develop hypertension, which is a significant risk factor for heart disease and stroke. Similarly, Hispanic/Latino populations also have higher rates of certain risk factors for CVD, such as obesity and diabetes. However, there is some variability within these subgroups, with individuals from Mexican and Puerto Rican backgrounds often exhibiting higher rates of CVD compared to those from Cuban or South American backgrounds [2,3]. Asian populations have traditionally had lower rates of CVD compared to Western populations, but there has been an increasing prevalence of CVD in many Asian countries due to changing lifestyles, including unhealthy dietary habits and reduced physical activity. Additionally, South Asians (people from countries like India, Pakistan, Bangladesh, etc.) tend to have higher rates of CVD compared to East Asians [4,5].

The Asian American population is one of the fastest-growing populations in the world, and it is estimated to reach approximately 34 million by the end of 2050 [4]. According to the recent AAPI demographic report for 2023, there are approximately 23.5 million Asian Americans, representing 7.1% of the US population. In combination with other races, 3.8 million people identify as Asian [5]. The Asian population comprises people from diverse ethnicities and backgrounds from different regions, mostly including China, India, Japan, the Philippines, Bangladesh, and Pakistan. In 2010, the American Heart Association initiated a call to highlight gaps in the cardiovascular risks involved within immigrant populations [4]. Cardiovascular disease prevalence is increasing globally, and the emerging data suggests more burden among South Asians who have high-risk factors, a younger age of onset, and worse outcomes when compared to the American population [6,7].

According to a case-control study conducted by INTERHEART in the early 2000s [8], involving 15,152 cases of acute myocardial infarction (AMI) and a total of 14,820 controls from 52 countries, 1732 patients and 2204 controls belonged to South Asian origin. The median age at first AMI was 63 for Chinese and Europeans, whereas 53 for South Asians. The highest proportions of patients with their first AMI at age 40 years of age or younger were men from the Middle East (12.6%), Africa (10.9%), and South Asia (9.7%), and the lowest proportions were in women from China and Hong Kong (1.2%), South America (1.0%), and Central and Eastern Europe (0.9%). These results [8] demonstrate the magnitude of the risk of premature coronary heart disease (CHD) in South Asia.

This paper presents a comprehensive examination of the multifaceted factors contributing to the variation in the prevalence of CVD among Asians (especially South Asians) and Americans. These further underscore the critical need for preventive measures and collaborative efforts across healthcare policy and educational domains. In the following sections, we will investigate the various contributors to early onset CVD, including genetic factors, dietary risks, sedentary lifestyles, and the imperative role of preventive measures. Additionally, we will discuss screening measures, pharmacotherapy options, and strategies for tackling this emerging public health crisis.

## Review

Methods

The study was conducted through a thorough literature review following the SANRA guidelines [9]. The research involved the search of academic databases such as PubMed, data reports from the American Heart Association, the World Health Federation, and other scientific journal publications from the past two decades.

Search Strategy and Selection criteria

Various factors, such as CVD prevalence, regional variations among Asians and Americans, dietary factors, lifestyle, and genetic heterogeneity, were considered in the present study. Age, sex analysis, and mortality rates were excluded. This study aims to provide a holistic perspective on the issue by analyzing selected articles rigorously. The goal was to synthesize existing knowledge and insights regarding the factors contributing to the increase in the prevalence of cardiovascular disease among South Asians, offering a comprehensive understanding of the problem's complexity.

The results of our analysis have been visually represented through Excel sheets in the form of graphs, offering a clear and informative perspective on the evolving trends in cardiovascular disease across different regions.

Results

**Figure 1:**
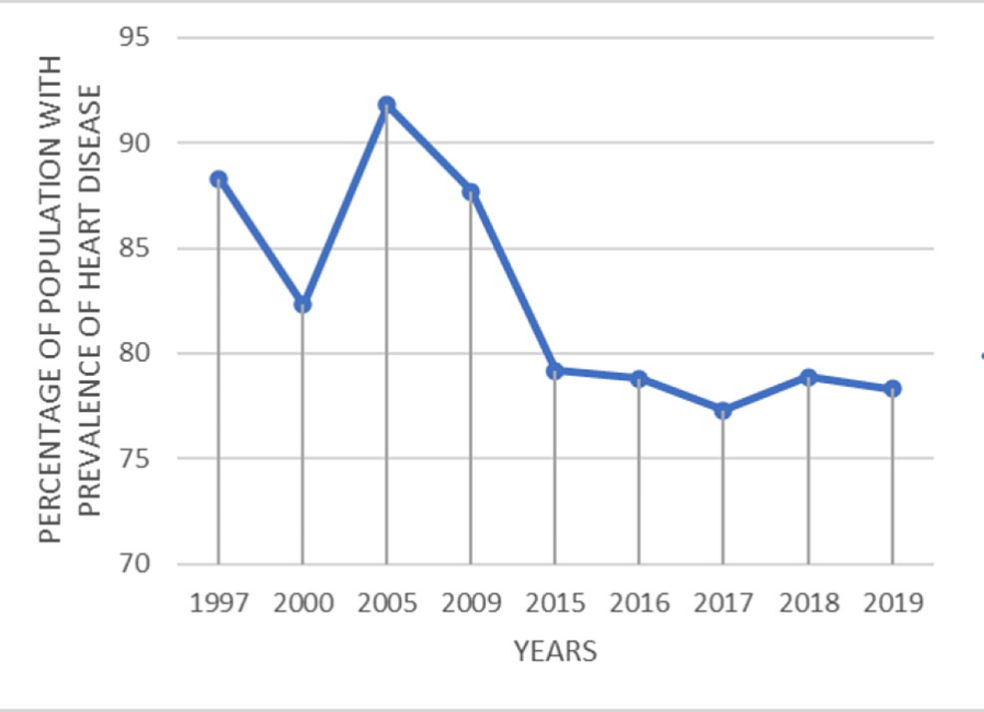
Trends in occurrence of heart disease in Americans This is an original graphical representation made by the authors, adapted from the National Center for Health Statistics (U.S.) [3].

**Figure 2:**
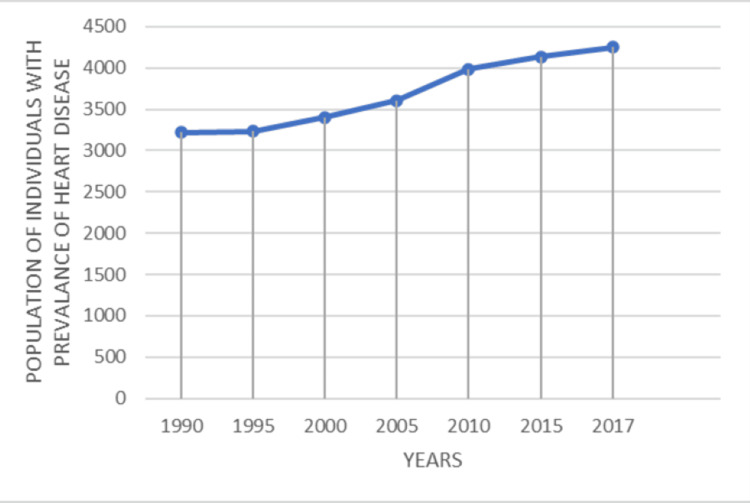
Trends in occurence of heart disease in Asians This is an original graphical representation made by the authors, adapted from the Trend analysis of cardiovascular disease mortality, incidence, and mortality-to-incidence ratio: results from the global burden of disease study 2017 [10].

The comparative data collected from cumulative reports carried out by Asian and American populations over the last two decades show that the number of cardiovascular disease cases in Asians has increased steadily. Although a sudden increase in the prevalence of heart disease occurred in 2005 in the U.S., the total prevalence decreased steadily throughout the years (Figures 1-2) [2,3,10].

Discussion

Genetic Heterogeneity

Certain genetic variants and polymorphisms are more common in South Asian populations and are associated with variable cholesterol levels - lower high-density lipoproteins and high low-density lipoproteins [11]. Genetic heterogeneity in APOA1 and APOB and variations in genes such as cholesteryl transfer ester protein (CETP) and hepatic lipase (LIPC) have also been linked to lower HDL levels [12]. The APO1 gene encodes the protein ApoA1, which is the main component of HDL cholesterol [13]. The absence of the APOA1 gene results in extremely low HDL-c cholesterol, which is evident in both human and mouse studies. The polymorphisms of the APO1 protein affect the HDL levels of an individual in different proportions. Hence, there is a strong correlation between low serum levels of APOA1 protein and the prevalence of heart disease. Given that lipid abnormalities represent a significant risk factor for cardiac health, any variations in APOA1 gene levels can directly influence the occurrence and progression of CVDs. The characterization of polymorphisms in the APOA1 gene has shed light on why South Asians are disproportionately affected by APOA1 protein levels. Therefore, APO1 protein levels are the single most powerful predictor of the development of heart disease. The University of Kansas Medical Center, in 2017 [14], studied South Asian immigrants, representing individuals from first and second generations living in the United States, for allele and genotype frequencies of single nucleotide proteins (SNPs). The results were then compared to the genome-wide variations among different countries. The study concludes that this variance in the APOA1 SNP pattern may contribute to the decreased levels of HDL cholesterol reported in South Asians, leading to an increased risk of developing cardiovascular disease, metabolic syndrome, and dysfunctional HDLs in this population. A similar study was conducted previously in 2012, wherein gene sequencing was done among a community of South Asian immigrants aged 35-65, and their genotype was compared to their cardiovascular findings. It was found that seven single nucleotide repeats of the APOA1 gene were different from those of Europeans. The study concludes that the discovery of novel polymorphisms will aid in understanding the risk factors in high-risk populations [15].

Polygenic Risk Score

Genetic variations are associated with a wide range of disease outcomes, but the effect of monogenic variations is effectively small. Hence, the researchers brought together a wide range of genetic variations and aggregated them into a single score. The polygenic risk score (PRS) for CVD quantifies the likelihood of developing the disease by calculating the presence or absence of genome-wide genetic variants that are associated with increased risk [16]. It is a numerical representation of an individual's genetic predisposition to developing coronary artery disease, hypertension, or stroke based on multiple genetic variants across the genome. In cardiovascular disease, various genetic variants across different chromosomes can contribute to an individual's overall risk. It was first published in 2010 in a study conducted by Ripatti et al. [17], where 13 single nucleotide variations (SNVs) repeats were studied in a case-control design among 52,726 participants and 30,725 participants in a prospective cohort design. Subsequently, numerous other studies explored a broad range of SNVs. Wang et al. studied the PRS among South Asian populations by analyzing >6 million common genetic variations that are associated with coronary artery disease. The study shows that the cumulative impact of PRS on cardiovascular disease is an important factor, even among South Asians. There is a significant difference in the risk ratio, and South Asians are more prone to cardiovascular disease when compared to Caucasians [18]. This study also establishes a new genome-wide polygenic score to provide a common framework for ancestry-specific assessment.

Dietary variance and lifestyle

Dyslipidemia

Traditional Asian diets consist of collections of a large variety of staple dietary additives such as spice pulses, vegetables, and whole grains, which are found to provide essentially good control of lipid levels and health protection [19]. Many metabolic diseases and age-related degenerative disorders, including cardiovascular disorders, are closely associated with oxidative processes in the body, and this is especially determined by the diet consumed by an individual. An individual's dietary habits have a varied influence on their HDL, LDL, and VLDL levels. These traditional diets are described as possessing medicinal properties, including antithrombotic, anti-atherosclerotic, hypolipidemic, hypoglycemic, anti-inflammatory, anti-arthritic, etc. However, recent changes in dietary habits with increased consumption of Western-style processed foods can negatively affect HDL “good” cholesterol levels. The consumption of a diet high in carbohydrates, including refined carbohydrates and saturated fats, can contribute to imbalances in cholesterol levels, including lower HDL cholesterol [20]. A recent study, the MASALA study [21], done in 2015, assessed the dietary patterns of South Asians using the Food Frequency Questionnaire. It showed that the animal protein diet is associated with increased BMI and abdominal circumferences, and the vegetarian diet, mainly consisting of fried snacks, dairy, and sweets, is associated with a rising prevalence of insulin resistance and lower HDL levels [22]. The use of different types of cooking oils also has varying effects on cholesterol profiles. Oils like canola, sesame, soya, olive, and sunflower oils have shown efficacy in lowering LDL levels and increasing HDL levels. These oils also contain vitamin E, providing protective effects against atherosclerosis. On the other hand, oils high in saturated fats, such as coconut oil, butter, trans fats, and palm oil, are associated with raising LDL levels, which can contribute to atherosclerosis [23]. A diet rich in unsaturated fats (such as those found in olive oil, avocados, and fatty fish), whole grains, fruits, vegetables, and nuts, which are staple foods for Americans, may contribute to their higher HDL levels [24]. Although most Indians are vegetarians, the majority of the population consumes large quantities of sugar and refined carbohydrates, which is problematic for this population. The INTERHEART study, conducted between 1999 and 2003, used a case-control design to compare the age- and sex-matched first cases of acute myocardial infarction and controls from 15 medical centers in 5 South Asian countries to the cases and controls from other countries. This study shows that higher HDL-C levels were associated with a decreased risk of acute myocardial infarction. However, the protective effect of higher HDL-C levels seemed to be weaker for South Asians than for other Asians [6]. It was also found that South Asians have higher risk factors at a very young age (<60 years). Another community-based cross-sectional study by Clara et al. assessed the correlation of cholesterol and diabetes with carotid intima-media thickness (CIMT) among South Asian Indians. Random samples from two rural Indian villages were taken for the assessment of cardiovascular risk factors and ultrasound-guided CIMT measurements (n = 303) and were compared to adult Caucasians from Australia (n = 1111). These studies showed that increasing HDL-C levels were associated with decreasing CIMT in the Australian population, whereas the reverse was true for the Indian population (P < 0.001) [25]. This proves that South Asians not only have low levels of HDL-C but also appear to have much less cardiovascular protection from HDL-C when compared to the other ethnic population.

Low-Density Lipoprotein

LDL cholesterol is often referred to as "bad" cholesterol because high levels of LDL can lead to the buildup of plaque in the arteries, a condition known as atherosclerosis. Atherosclerosis is a major risk factor for cardiovascular diseases, including coronary artery disease, heart attack, and stroke. Regular monitoring of LDL cholesterol levels and overall cardiovascular risk assessment are important for individuals at risk of cardiovascular disease. The elevated LDL-C levels in South Asians indicate a greater risk of CVD than those of Americans. Few studies have shown that levels of LDL-C are similar among South Asian and non-Asian populations [20], but South Asians are more prone to CVD because of the presence of greater particle burden and lower particle density at the same given LDL-C level [21]. They have also shown that the status of insulin resistance, abdominal obesity, and LDL particle concentrations modulate the risk of CVD significantly. A case-control study done across 65 centers in Asia on the first cases of acute MI and controls showed that for any LDL-C level, South Asians had higher apolipoprotein (apo) B concentrations than did any other ethnic population, indicating that for any given LDL-C level, South Asians carry a larger number of atherogenic lipoproteins [20]. This study also shows that the HDL-c levels among South Asians, when compared to the rest of Asia, are substantially lower (p<0.0001). The triglyceride levels are also low compared to people from China and Hong Kong. The South Asians had the highest ApoB levels, and the Chinese had the lowest levels in comparison to the other Asian subgroups, indicating a larger atherogenic particle load and a smaller particle size among the South Asians.

Lifestyle

A sedentary lifestyle, characterized by low levels of physical activity and prolonged periods of sitting or inactivity, has significant negative effects on cardiovascular health due to its effects on obesity and weight gain, poor lipid profile, hypertension, insulin resistance and diabetes, inflammation, and oxidative stress. It is a significant modifiable risk factor for cardiovascular disease. Engaging in regular physical activity and reducing sedentary behavior is crucial for maintaining cardiovascular health and reducing the risk of heart disease and stroke. In a cross-sectional study, data from 1038 Indians settled in the US were collected to investigate the relationship between metabolic syndrome, cardiovascular disease, and type 2 diabetes. It is found that reduced physical activity, lack of awareness, and advanced age are significantly associated with low HDL levels [22]. It also shows that a more sedentary lifestyle is likely to be seen in Asian Indians living in the US for more than 10 years. The Health of Houston survey of 2010 reports that the rate of physical activity among Asians is far lower compared to other ethnic groups [23]. Factors such as language, age, immigration status, marital status, and neighborhood can significantly affect physical activity levels. Another cross-sectional study between the New York City Health Service and the Los Angeles County Health Survey shows that Asian Americans have a low prevalence of achieving physical activity goals, as stated by the American Heart Association [24]. Studies have also shown that social norms, traditional methods of eating, gender-specific gyms, and a lack of time could be possible barriers to achieving optimal physical activity goals [26,27].

Both smoking and excessive alcohol consumption are well-established risk factors for CVD. Higher rates of smoking and alcohol consumption can also negatively affect HDL levels. In a cross-sectional study conducted on Japanese men and women, the association of HDL-p/c with lifestyle was investigated [28]. It has been found that consumption of alcohol is associated with large-size HDL particles, which are strongly associated with CAD and subclinical atherosclerosis. On the other hand, smoking is associated with reduced large-size HDL particles, but given the oxidative effects of tobacco, it oxidizes HDL, leading to atherosclerosis.

Vitamin D Deficiency

In the last decade, new risk factors for cardiovascular diseases have been identified, and vitamin D deficiency is one of them. It is an emerging epidemic worldwide, and the relationship between vitamin D and CVD is a topic of ongoing research and debate. An estimated of around billion people all over the world have low vitamin D levels [29]. Some research suggests that adequate vitamin D levels may help regulate blood pressure, which is a key risk factor for CVD. Vitamin D may influence the renin-angiotensin-aldosterone system, which plays a role in blood pressure regulation [30]. Hypovitaminosis has been recently studied to cause an increase in the all-cause mortality rate, especially in deaths due to cardiovascular diseases. It was found to be associated with patients with heart failure, myocardial infarction, and peripheral artery disease [31]. A drop in vitamin D levels is found to have a significant association with vascular diseases and heart failure in the US population [32]. A similar association is also seen among the Asian population. Low serum vitamin D levels are associated with obesity, diabetes, and cardiometabolic diseases [33].

Preventive measures for CVD

Although heart disease is typically linked to older age, early preventive measures greatly lower CVD risk. This involves a range of strategies and interventions, from early prevention to later stages. A healthy diet, regular exercise, maintenance of a healthy weight, quitting smoking and limited alcohol consumption, stress management, blood pressure, cholesterol, and blood sugar control are some of the modifiable risk factors. Maximizing effectiveness means applying these measures at all stages, emphasizing healthy living, addressing risk factors, and offering proper care for those with CVD.

**Figure 3:**
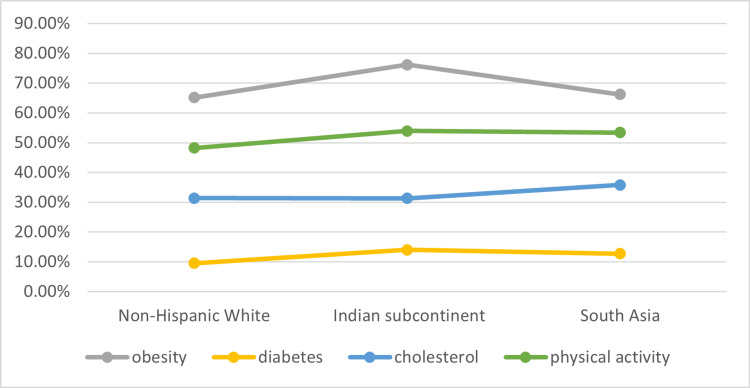
Age adjusted prevalence of CVD risk factors This is an original graphical representation made by the authors, adapted from data from the 2010–2018 National Health Interview Survey [34].

The below analysis shows the data from a cross-sectional analysis reported by the National Health Interview Survey among the individuals born in Asian regions and those Hispanic white or the ones born in the US [34] (Figure 3). It shows that diabetes and obesity are proportionally greater in individuals from the Indian subcontinent, whereas dyslipidemia and abnormal cholesterol levels are higher throughout South Asia. Reduced physical activity is almost similar among Indians and the rest of South Asians. The non-Hispanic Whites show significantly lower levels of CVD risk factors.

Optimal lipid levels are crucial for heart health and can be achieved through a nutritious diet [35]. HDL cholesterol is just one component of total cholesterol, and maintaining the right balance between cholesterol, HDL, LDL, and VLDL is essential. Asian Indians have higher plasma triglycerides but lower HDL-C than Americans, while Americans have higher total cholesterol, non-HDL-C, and LDL-C [36]. To balance lipids, increasing the intake of unsaturated fats from plant foods, fruits, nuts, and spices must be prioritized, and care must be taken to minimize the consumption of saturated fats from Western and fast foods. A modified balanced dietary recommendation, the DASH diet, and the Mediterranean diet can eventually reduce the risk burden of cardiovascular disease, along with positive effects on hypertension and diabetes control [37].

Genetic screening for CVD involves analyzing an individual's genetic makeup to identify specific genetic variants associated with an increased risk of developing heart disease. While genetic screening can provide valuable insights into an individual's susceptibility to CVD, it is important to note that genetics is just one component of overall cardiovascular risk, and lifestyle and environmental factors also play significant roles. Genetic screening is vital for preventing congenital heart disease, especially in individuals with a family history. Identifying the genetic basis of APOB proteins enables early detection and personalized interventions to reduce cardiovascular disease [38]. It is also valuable for identifying individuals at risk of inherited forms of cardiovascular disease, such as familial hypercholesterolemia or hypertrophic cardiomyopathy. Family screening and genetic counseling can help identify at-risk family members and provide guidance on preventive measures and screening protocols.

Regular physical activity offers cardiovascular benefits. The American Heart Association guidelines recommend gradually increasing 150-300 minutes of moderate-intensity or 75-150 minutes of vigorous-intensity aerobic activity per week. Muscle-strengthening exercises were performed at least twice a week [39]. One should aim for at least 150 minutes of moderate-intensity aerobic activity or 75 minutes of vigorous-intensity aerobic activity per week. Awareness must be raised by organizing culturally appropriate interventions to promote physical activity in Asian American communities.

Hypertension, diabetes, and chronic kidney disease can lead to cardiovascular issues. In a recent study using data from 2011 to 2014, the National Health and Nutrition Examination Survey revealed a greater prevalence of diabetes mellitus and obesity among the foreign-born Asian population than among the non-Hispanic white population [40]. Another study reported that the prevalence of type 2 diabetes mellitus among adults in the United States, divided by race or ethnicity, was 27% in South Asian immigrants as compared to 8.0% in the non-Hispanic white population [41]. Regular check-ups and appropriate medication can limit this risk. Adolescents should have their blood pressure checked annually, and young adults should monitor it at every visit, as many may be unaware of hypertension. A healthy diet with salt restriction should also be pursued for optimal blood pressure control [42]. Quitting smoking and moderating alcohol intake are also important steps individuals can take to reduce their risk of heart disease, stroke, and other cardiovascular conditions. Individuals should also consult with their healthcare providers regarding vitamin D supplementation and its potential role in their cardiovascular health management.

Pharmaceutical measures

Pharmacological options for preventing cardiovascular disease in younger adults with high lifetime risk may include statins, blood pressure-lowering drugs, and newer treatments such as PCSK9 inhibitors and newer antidiabetic medications. These agents can be studied alone or in combination with other interventions [43]. Statins are a class of medications used to lower LDL (bad) cholesterol levels in the blood, thus slowing down the progression of atherosclerosis and reducing the risk of cardiovascular events such as heart attack and stroke. Statins also have anti-inflammatory effects that contribute to their cardiovascular benefits. Statins are approved for use in adolescent boys and postpubertal girls. At the same time, anti-obesity medications are generally off-label for young individuals, except for orlistat (approved for 12 years and older) and phentermine (approved for 16 years and older) [44].

Different classes of antihypertensive drugs, such as ACE inhibitors, angiotensin II receptor blockers (ARBs), beta-blockers, calcium channel blockers (CCBs), and diuretics, may be prescribed based on individual patient characteristics and comorbidities. In adolescents without diabetes or renal disease, CCBs are suitable for hypertension treatment. In contrast, those at risk of diabetes and kidney disease can benefit from angiotensin-converting enzyme inhibitors (ACEi) and angiotensin receptor blockers (ARBs).

Anticoagulant medications, such as warfarin or direct oral anticoagulants (DOACs) like apixaban, dabigatran, and rivaroxaban, are used to prevent blood clot formation and reduce the risk of stroke in individuals with atrial fibrillation.

For individuals with diabetes, medications such as metformin, sulfonylureas, DPP-4 inhibitors, GLP-1 receptor agonists, SGLT2 inhibitors, and insulin may be prescribed to manage blood sugar levels and reduce the risk of cardiovascular complications associated with diabetes. According to the updated guidelines of the American Diabetic Association, patients with T2DM who are diagnosed with CVD or have risk factors for CVD can use GLP-1RAs and SGLT2 inhibitors, which have evidence of cardiovascular benefits, regardless of HbA1c levels [44].

Screening measures

Screening measures for the prevention of CVD in Asians are similar to those recommended for other populations but may need to be tailored to address specific risk factors and considerations that are more prevalent among Asian populations. Asians, including Indians, may benefit from genetic screening for specific CVD risk factors, such as familial hypercholesterolemia or genetic predispositions to hypertension. Genetic testing can help identify individuals at greater risk. Hypertension was defined as blood pressure above the 95th percentile for age, sex, and height. Asian populations may have a predisposition to develop hypertension at lower blood pressure levels compared to other ethnic groups. Therefore, screening should take into account lower blood pressure thresholds for diagnosing hypertension in Asians. In young adults, it is confirmed whether readings are consistently measured at ≥140/90 on two occasions [45]. Early detection and treatment of indicators such as albuminuria, proteinuria, GFR, and creatinine are crucial for hypertension and type 2 diabetes patients. Fasting blood glucose, oral glucose tolerance tests (OGTT), or HbA1c tests may be used for diabetes screening, with consideration for lower diagnostic thresholds in Asians. Screening for type 2 diabetes is recommended for overweight individuals with risk factors [46]. Healthy lifestyles can be promoted through school programs, marathons, and technology-driven campaigns. Asians may have a higher proportion of body fat and an increased risk of central obesity at lower BMI levels compared to other ethnic groups. Therefore, lower BMI thresholds may be used to define overweight and obesity in Asians.

Electrocardiograms and stress tests were used to assess abnormal heart rhythms, hypertrophy, and cardiac issues in those with chest pain. Coronary artery calcium (CAC) measures coronary artery calcium and indicates atherosclerosis risk. CAC scores of 100-400, 401-999, and 1000+ corresponded to relative risks of 4.3, 7.2, and 10.8, respectively [47]. Blood pressure in the arms and ankles in the ankle-brachial index (ABI) test for peripheral artery disease. At the same time, carotid ultrasound detects atherosclerotic plaques in carotid arteries, increasing stroke and heart disease risk.

Healthcare providers need to consider ethnic-specific risk factors and screening guidelines when evaluating cardiovascular risk in Asian individuals. Additionally, lifestyle modifications and preventive interventions should be tailored to address the specific needs and risk profiles of Asian populations. Regular health check-ups and screenings, along with lifestyle modifications, remain key components of cardiovascular disease prevention in Asians and other populations.

Limitations

The study included data up to 2019 from American population statistics and 2017 from Asian population reports. The study does not include data from recent studies, which may have led to a significant bias in the results. In addition, we did not include data on variations in age, sex, or mortality rates due to CVD, which could have significantly altered the course of the paper. The paper does not discuss the variations in laboratory values and guidelines that need to be adapted while stratifying the risk factor threshold levels among different populations. It is beyond the scope of the paper, and relevant studies and further research have to be conducted to be able to adapt standard ethnicity-based risk calculation guidelines.

## Conclusions

This study illuminates the stark differences in CVD prevalence between Asians and Americans, shedding light on the intricate interplay of genetics, diet, and lifestyle in shaping these disparities. Fundamentally, this finding underscores the need for an ethnicity-oriented approach to address these challenges effectively. The genetic diversity within the Asian population itself calls for early genetic screening and personalized interventions to mitigate CVD risk effectively. Similarly, culturally sensitive dietary interventions and public health initiatives should emphasize the preservation of traditional dietary practices while promoting the adoption of healthier choices.

In conclusion, addressing the increasing prevalence of CVD in Asians requires a deep understanding of the region's unique genetic makeup, dietary habits, and cultural factors. The implementation of tailored prevention strategies, culturally sensitive education, and proactive screening measures are essential to combat this global health challenge effectively among all populations.

## References

[1] (2024). Department of Data and Analytics (DNA) Division of Data A and D for I (DDI) WG. WHO methods and data sources for country-level causes of death 2000-2019. https://www.who.int/data/gho/data/themes/mortality-and-global-health-estimates/ghe-leading-causes-of-death.

[2] Tsao CW, Aday AW, Almarzooq ZI (2023). Heart disease and Stroke Statistics-2023 update: a report from the American Heart Association. Circulation.

[3] National Center for Health Statistics (US) (2024). Health, United States, Annual Perspective, 2020-2021. https://www.cdc.gov/nchs/hus/report.htm.

[4] Palaniappan LP, Araneta MR, Assimes TL (2010). Call to action: cardiovascular disease in Asian Americans: a science advisory from the American Heart Association. Circulation.

[5] (2023). AAPI demographics: Data on Asian American ethnicities, geography, income, and education. https://usafacts.org/articles/the-diverse-demographics-of-asian-americans/.

[6] Martinez-Amezcua P, Haque W, Khera R (2020). The upcoming epidemic of heart failure in South Asia. Circ Heart Fail.

[7] Harikrishnan S, Jeemon P, Ganapathi S (2021). Five-year mortality and readmission rates in patients with heart failure in India: Results from the Trivandrum heart failure registry. Int J Cardiol.

[8] Joshi P, Islam S, Pais P (2007). Risk factors for early myocardial infarction in South Asians compared with individuals in other countries. JAMA.

[9] Baethge C, Goldbeck-Wood S, Mertens S (2019). SANRA-a scale for the quality assessment of narrative review articles. Res Integr Peer Rev.

[10] Amini M, Zayeri F, Salehi M (2021). Trend analysis of cardiovascular disease mortality, incidence, and mortality-to-incidence ratio: results from global burden of disease study 2017. BMC Public Health.

[11] Bilen O, Kamal A, Virani SS (2016). Lipoprotein abnormalities in South Asians and its association with cardiovascular disease: Current state and future directions. World J Cardiol.

[12] Weissglas-Volkov D, Pajukanta P (2010). Genetic causes of high and low serum HDL-cholesterol. J Lipid Res.

[13] Kamboh MI, Aston CE, Nestlerode CM, McAllister AE, Hamman RF (1996). Haplotype analysis of two APOA1/MspI polymorphisms in relation to plasma levels of apo A-I and HDL-cholesterol. Atherosclerosis.

[14] Henkhaus RS, Dodani S, Manzardo AM, Butler MG (2011). APOA1 gene polymorphisms in the South Asian immigrant population in the United States. Indian J Hum Genet.

[15] Dodani S, Henkhaus R, Dong L, Butler MG (2012). Apo lipoprotein A1 gene polymorphisms predict cardio-metabolic risk in South Asian immigrants. Dis Markers.

[16] O'Sullivan JW, Raghavan S, Marquez-Luna C (2022). Polygenic risk scores for cardiovascular disease: a scientific statement from the American Heart Association. Circulation.

[17] Ripatti S, Tikkanen E, Orho-Melander M (2010). A multilocus genetic risk score for coronary heart disease: case-control and prospective cohort analyses. Lancet.

[18] Wang M, Menon R, Mishra S (2020). Validation of a genome-wide polygenic score for coronary artery disease in South Asians. J Am Coll Cardiol.

[19] Vasanthi HR, Parameswari RP (2010). Indian spices for healthy heart - an overview. Curr Cardiol Rev.

[20] Karthikeyan G, Teo KK, Islam S (2009). Lipid profile, plasma apolipoproteins, and risk of a first myocardial infarction among Asians: an analysis from the INTERHEART Study. J Am Coll Cardiol.

[21] Lamarche B, Lemieux I, Després JP (1999). The small, dense LDL phenotype and the risk of coronary heart disease: epidemiology, patho-physiology and therapeutic aspects. Diabetes Metab.

[22] Lucke-Wold B, Misra R, Patel TG (2017). Risk factors for low high-density lipoprotein among Asian Indians in the United States. World J Diabetes.

[23] Kao D, Carvalho Gulati A, Lee RE (2016). Physical activity among Asian American adults in Houston, Texas: data from the health of Houston survey 2010. J Immigr Minor Health.

[24] Yi SS, Roberts C, Lightstone AS, Shih M, Trinh-Shevrin C (2015). Disparities in meeting physical activity guidelines for Asian-Americans in two metropolitan areas in the United States. Ann Epidemiol.

[25] Chow CK, McQuillan B, Raju PK (2008). Greater adverse effects of cholesterol and diabetes on carotid intima-media thickness in South Asian Indians: comparison of risk factor-IMT associations in two population-based surveys. Atherosclerosis.

[26] Farooqi A, Nagra D, Edgar T, Khunti K (2000). Attitudes to lifestyle risk factors for coronary heart disease amongst South Asians in Leicester: a focus group study. Fam Pract.

[27] Galdas PM, Oliffe JL, Wong ST, Ratner PA, Johnson JL, Kelly MT (2012). Canadian Punjabi Sikh men's experiences of lifestyle changes following myocardial infarction: cultural connections. Ethn Health.

[28] Zaid M, Miura K, Okayama A (2018). Associations of high-density lipoprotein particle and high-density lipoprotein cholesterol with alcohol intake, smoking, and body mass index - the INTERLIPID study. Circ J.

[29] Holick MF, Chen TC (2008). Vitamin D deficiency: a worldwide problem with health consequences. Am J Clin Nutr.

[30] Adams JS, Hewison M (2010). Update in vitamin D. J Clin Endocrinol Metab.

[31] Artaza JN, Mehrotra R, Norris KC (2009). Vitamin D and the cardiovascular system. Clin J Am Soc Nephrol.

[32] Kim DH, Sabour S, Sagar UN, Adams S, Whellan DJ (2008). Prevalence of hypovitaminosis D in cardiovascular diseases (from the National Health and Nutrition Examination Survey 2001 to 2004). Am J Cardiol.

[33] Lu YW, Chou RH, Liu LK, Chen LK, Huang PH, Lin SJ (2020). The relationship between circulating vitamin D3 and subclinical atherosclerosis in an elderly Asian population. Sci Rep.

[34] Koirala B, Turkson-Ocran RA, Baptiste D (2021). Heterogeneity of cardiovascular disease risk factors among Asian immigrants: insights from the 2010 to 2018 National Health Interview Survey. J Am Heart Assoc.

[35] Szczepańska E, Białek-Dratwa A, Janota B, Kowalski O (2022). Dietary therapy in prevention of cardiovascular disease (CVD)-tradition or modernity? A review of the latest approaches to nutrition in CVD. Nutrients.

[36] Singh K, Thanassoulis G, Dufresne L (2021). A comparison of lipids and ApoB in Asian Indians and Americans. Glob Heart.

[37] Zhang N, Xiao X, Xu J (2022). Dietary approaches to stop hypertension (DASH) diet, Mediterranean diet and blood lipid profiles in less-developed ethnic minority regions. Br J Nutr.

[38] Sniderman AD, Thanassoulis G, Glavinovic T, Navar AM, Pencina M, Catapano A, Ference BA (2019). Apolipoprotein B particles and cardiovascular disease: a narrative review. JAMA Cardiol.

[39] Piercy KL, Troiano RP, Ballard RM (2018). The Physical Activity Guidelines for Americans. JAMA.

[40] Echeverria SE, Mustafa M, Pentakota SR, Kim S, Hastings KG, Amadi C, Palaniappan L (2017). Social and clinically-relevant cardiovascular risk factors in Asian Americans adults: NHANES 2011-2014. Prev Med.

[41] Cheng YJ, Kanaya AM, Araneta MR (2019). Prevalence of diabetes by race and ethnicity in the United States, 2011-2016. JAMA.

[42] Taylor RS, Ashton KE, Moxham T, Hooper L, Ebrahim S (2011). Reduced dietary salt for the prevention of cardiovascular disease. Cochrane Database Syst Rev.

[43] Navar AM, Fine LJ, Ambrosius WT (2022). Earlier treatment in adults with high lifetime risk of cardiovascular diseases: What prevention trials are feasible and could change clinical practice? Report of a National Heart, Lung, and Blood Institute (NHLBI) Workshop. Am J Prev Cardiol.

[44] (2022). Standards of care in diabetes-2023 abridged for primary care providers. Clin Diabetes.

[45] Chung RJ, Touloumtzis C, Gooding H (2015). Staying young at heart: cardiovascular disease prevention in adolescents and young adults. Curr Treat Options Cardiovasc Med.

[46] (2011). Expert panel on integrated guidelines for cardiovascular health and risk reduction in children and adolescents: summary report. Pediatrics.

[47] Neves PO, Andrade J, Monção H (2017). Coronary artery calcium score: current status. Radiol Bras.

